# Design of High Performance Si/SiGe Heterojunction Tunneling FETs with a T-Shaped Gate

**DOI:** 10.1186/s11671-017-1958-3

**Published:** 2017-03-16

**Authors:** Wei Li, Hongxia Liu, Shulong Wang, Shupeng Chen, Zhaonian Yang

**Affiliations:** 1 Key Laboratory for Wide Band Gap Semiconductor Materials and Devices of Education, School of Microelectronics, Xidian University, Xi’an, 710071 China; 2 School of Automation and Information Engineering, Xi’an University of Technology, Xi’an, 710048 China

**Keywords:** Band-to-band tunneling (BTBT), T-shaped gate, Tunneling field-effect transistor (TFET), Heterojunction

## Abstract

In this paper, a new Si/SiGe heterojunction tunneling field-effect transistor with a T-shaped gate (HTG-TFET) is proposed and investigated by Silvaco-Atlas simulation. The two source regions of the HTG-TFET are placed on both sides of the gate to increase the tunneling area. The T-shaped gate is designed to overlap with N^+^ pockets in both the lateral and vertical directions, which increases the electric field and tunneling rate at the top of tunneling junctions. Moreover, using SiGe in the pocket regions leads to the smaller tunneling distance. Therefore, the proposed HTG-TFET can obtain the higher on-state current. The simulation results show that on-state current of HTG-TFET is increased by one order of magnitude compared with that of the silicon-based counterparts. The average subthreshold swing (SS) of HTG-TFET is 44.64 mV/dec when *V*_g_ is varied from 0.1 to 0.4 V, and the point SS is 36.59 mV/dec at *V*_g_ = 0.2 V. Besides, this design cannot bring the sever Miller capacitance for the TFET circuit design. By using the T-shaped gate and SiGe pocket regions, the overall performance of the TFET is optimized.

## Background

Tunneling field-effect transistor (TFET) has become a kind of potential electric device for the ultralow power consumption applications [1–3]. Because band-to-band tunneling (BTBT) is the main operation mechanism in TFETs, TFETs can break the limitation of 60 mV/dec subthreshold swing (SS) in the conventional CMOS field-effect transistor that relies on the hot electron emission [4–6]. In addition, TFETs are less influenced by short channel effects than MOSFETs. However, the low on-state current is an inherent disadvantage in the traditional TFETs. In order to improve the on-state current of TFETs, various novel device structures have been proposed such as L-shaped channel TFET (LTFET) [7, 8], U-shaped channel TFET (UTFET) [9], L-shaped gate TFET (LG-TFET) [10], heterojunction TFET (HTFET) [11, 12]. Among these structures, the LG-TFET is proved to be essential for the enhancement of on-state current, because its tunneling current mainly depends on the electron BTBT perpendicular to the channel instead of parallel to the channel, and gate-pocket overlap regions in the lateral direction increase the electric field at the top of tunneling junction, which is helpful for the improvement of on-state current [10, 13, 14]. But electron BTBT in LG-TFET occurs only on one side of the gate, which will limit further improvement of on-state current.

In order to solve the above problem, a new heterojunction TFET with a T-shaped gate (HTG-TFET) is proposed. The proposed device structure remains vertical tunneling and places two source regions on both sides of the gate to further increase tunneling area. The T-shaped gate overlaps with the pocket regions in the lateral direction to increase the electric field at the top of tunneling junction. In addition, the heterojunctions between SiGe pocket regions and silicon source regions promote energy band to bend sharply [15]. TCAD simulation results show that proposed HTG-TFET gains higher on-state current and lower SS than both LG-TFET and UTFET.

## Methods

**Fig. 1:**
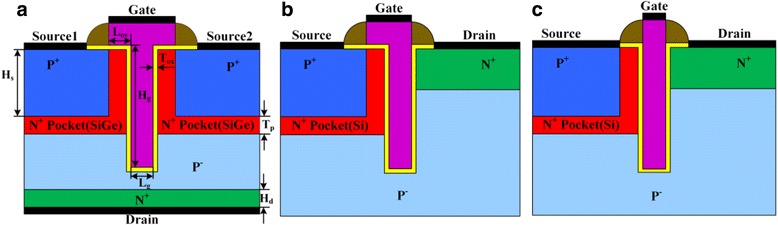
Schematic structures of **a** HTG-TFET, **b** LG-TFET, and **c** UTFET

**Fig. 2:**
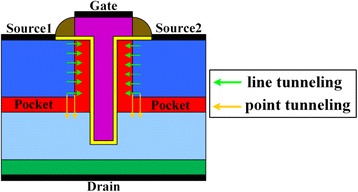
Schematic of line tunneling and point tunneling in HTG-TFET

The HTG-TFET discussed in this paper is illustrated in Fig. 1a. Compared with the conventional planar TFET, the HTG-TFET uses the recessed channel on the substrate to transform point tunneling parallel to channel into line tunneling perpendicular to channel, which increases the tunneling area and on-state current. Unlike the LG-TFET and UTFET shown in Fig. 1b, c, the HTG-TFET applies the dual sources to increase the tunneling area and its drain is placed at the bottom of the devices to decrease the gate-drain capacitance (*C*_*gd*_). Its gate overlaps with N^+^ pockets in both the vertical and the lateral directions. Therefore, the gate of HTG-TFET resembles the alphabet “T.” As shown in Fig. 2, using this structure, both the line tunneling and the point tunneling simultaneously take place on both sides of gate, which can enhance on-state current. In addition, the gate-pocket overlap in the lateral direction increases the electric field and tunneling area at the top of tunneling junction when the device is turned on. Since high electric field can induce higher BTBT generation rate, the overlap is helpful for the enhancement of the on-state current [9]. There is a lot of work which has demonstrated that heterojunction TFETs consisted of Si, Ge, and SiGe alloys are considered the most promising material system for the TFET due to their natural abundance and well-established fabrication technology; therefore, the pocket regions choose the narrow bandgap SiGe instead of silicon, which decreases tunneling distance to boost on-state current [16].

The proposed HTG-TFET structure is investigated with Silvaco ATLAS simulation tool using the non-local BTBT model. The non-local BTBT model takes into account the spatial variation of the energy band, and it also considers that the generation/recombination of the opposite carrier type is not spatially coincident. So, the non-local BTBT can model the tunneling process more accurately [17]. A lot of work has demonstrated that TFETs simulated by non-local BTBT are in accord with the experiments [8, 10, 18]. Since the source regions are highly doped, the band gap narrowing model and Fermi-Dirac statistics are included. The Shockley-Read-Hall recombination and Lombardi mobility models are also adopted in the simulations. Moreover, the gate leakage current is ignored.

The simulation parameters of the proposed device are as follows: thickness of the N^+^ pocket is 5 nm (*T*_p_); height of the source regions and drain region is 40 nm (*H*_s_) and 20 nm (*H*_d_), respectively; length and height of the gate are 10 nm (*L*_g_) and 60 nm (*H*_g_), respectively; thickness of the gate oxide (HfO_2_) is 2 nm (*T*_ox_); length of gate-pocket overlap is 7 nm (*L*_ov_). What is more, gate work function *φ* is 4.33 eV; doping concentrations of P^+^ source regions (*N*_s_) and N^+^ drain region (*N*_d_) are 1 × 10^20^/cm^3^ and 1 × 10^18^/cm^3^, respectively; doping concentration of N^+^ pockets (N_p_) is 1 × 10^19^/cm^3^.

## Results and Discussion

**Fig. 3:**
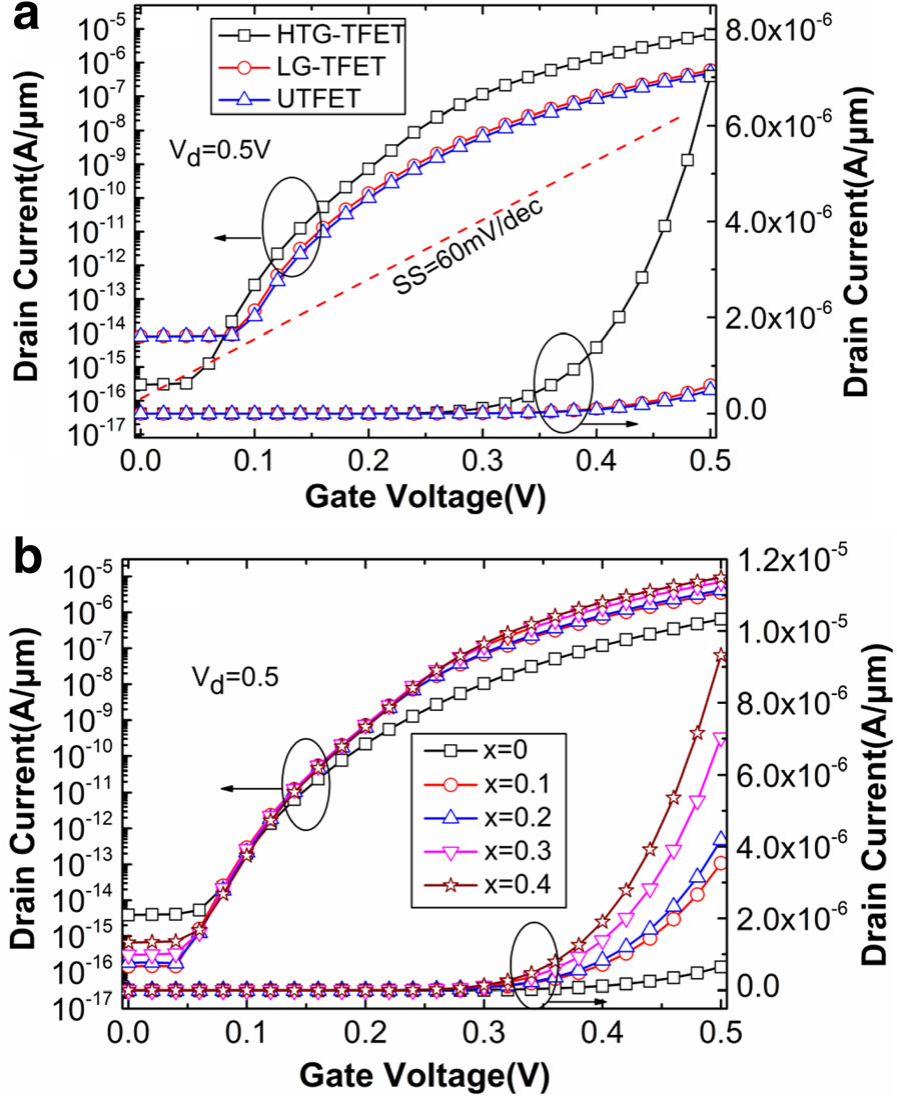
**a** Transfer characteristics of different devices. **b** Transfer characteristics with different Ge compositions for the HTG-TFET

Figure 3a shows the transfer characteristics of HTG-TFET, LG-TFET, and UTFET at *V*_d_ = 0.5 V. It can be seen from Fig. 3a that HTG-TFET has the largest on-state (*V*_d_ = *V*_g_ = 0.5 V) drain current and the smallest off-state (*V*_d_ = 0.5 V, *V*_g_ = 0 V) drain current due to the improved techniques (dual sources, T-shaped gate, Si/SiGe heterojunction). Compared with the LG-TFET and UTFET, the on-state and off-state drain currents of the HTG-TFET are increased and decreased by about one order of magnitude, respectively. Figure 3a also clearly shows that the average SSs of these three devices are less than 60 mV/dec which is indicated by red dotted line in Fig. 3a. The HTG-TFET obtains the minimum average SS and point SS. The average SSs extracted from 0.1 to 0.4 V are HTG-TFET = 44.64 mV/dec, LG-TFET = 47.21 mV/dec, and UTFET = 46.65 mV/dec; and the point SSs (at *V*g = 0.2 V) are HTG-TFET = 36.59 mV/dec, LG-TFET = 46.99 mV/dec, and UTFET = 45.52 mV/dec. The influence of Ge composition in SiGe pocket on the performance of HTG-TFET is shown in Fig. 3b. Obviously, both on-state and off-state currents increase when the Ge composition increases from 0.1 to 0.4. This is due to the fact that band gap of Si_1−x_Ge_x_ decreases with increasing Ge composition. Since the highest Ge composition of Si_1−x_Ge_x_ is around 40% in industrial production [9], the Ge composition *x* = 0.3 is regarded as the optimal Ge composition parameter in all the simulations. And Si_0.7_Ge_0.3_ pocket can help HTG-TFET to obtain the not only higher on-state current but also lower off-state current.

**Fig. 4:**
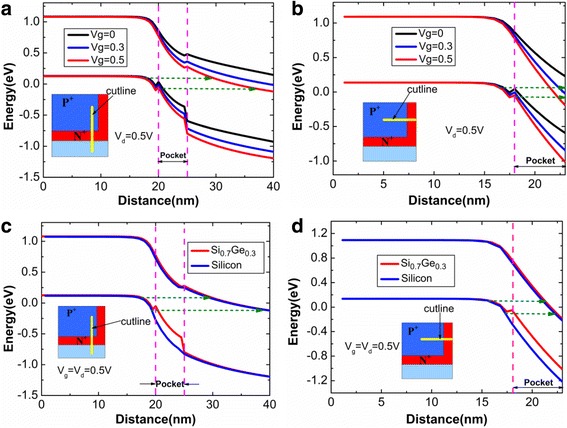
The influence of gate voltage on **a** point tunneling and **b** line tunneling energy band diagrams; **c** point tunneling and **d** line tunneling energy band diagrams of HTG-TFET with different pocket materials

In order to clearly understand the working mechanism of devices, the energy band diagrams are displayed in Fig. 4 (the inset in the figure shows the location of cutline). From Fig. 4a, b, it can be clearly seen that both point tunneling and line tunneling widths (green dot arrow) are very larger at *V*g = 0 V, which makes valance band of source region unable to align with conduction band of pocket so that the off-state current is very low. When the gate voltage rises to 0.3 V and it continues to increase, the tunneling widths obviously decrease due to the sharp bending of energy band. As a result, the drain current increases with the increasing gate voltage. Fortunately, the smaller valence band offset at Si/Si_0.7_Ge_0.3_ is found, which does not have a negative effect on device performance. Considering the bandgap narrowing effect caused by heavy doping, the bandgaps of Si and Si_0.7_Ge_0.3_ are respectively reduced to 0.96 and 0.82 eV. Besides, the heavily doped Si_0.7_Ge_0.3_ pocket makes the energy band of Si_0.7_Ge_0.3_ to drop down significantly. Combining the bandgap narrowing effect and heavily doped pocket, such a small valence offset does not create a large barrier to suppress electron to tunnel towards conduction band from valence band. Comparing the energy bands of devices with different pocket materials in Fig. 4c, d, it can be found that tunneling width with Si_0.7_Ge_0.3_ pocket is a lot smaller than that that with Si pocket, which is mainly caused by the smaller bandgap of Si_0.7_Ge_0.3_. Therefore, the HTG-TFET with Si_0.7_Ge_0.3_ pocket can obtain more superior performance than that with silicon pocket.

**Fig. 5:**
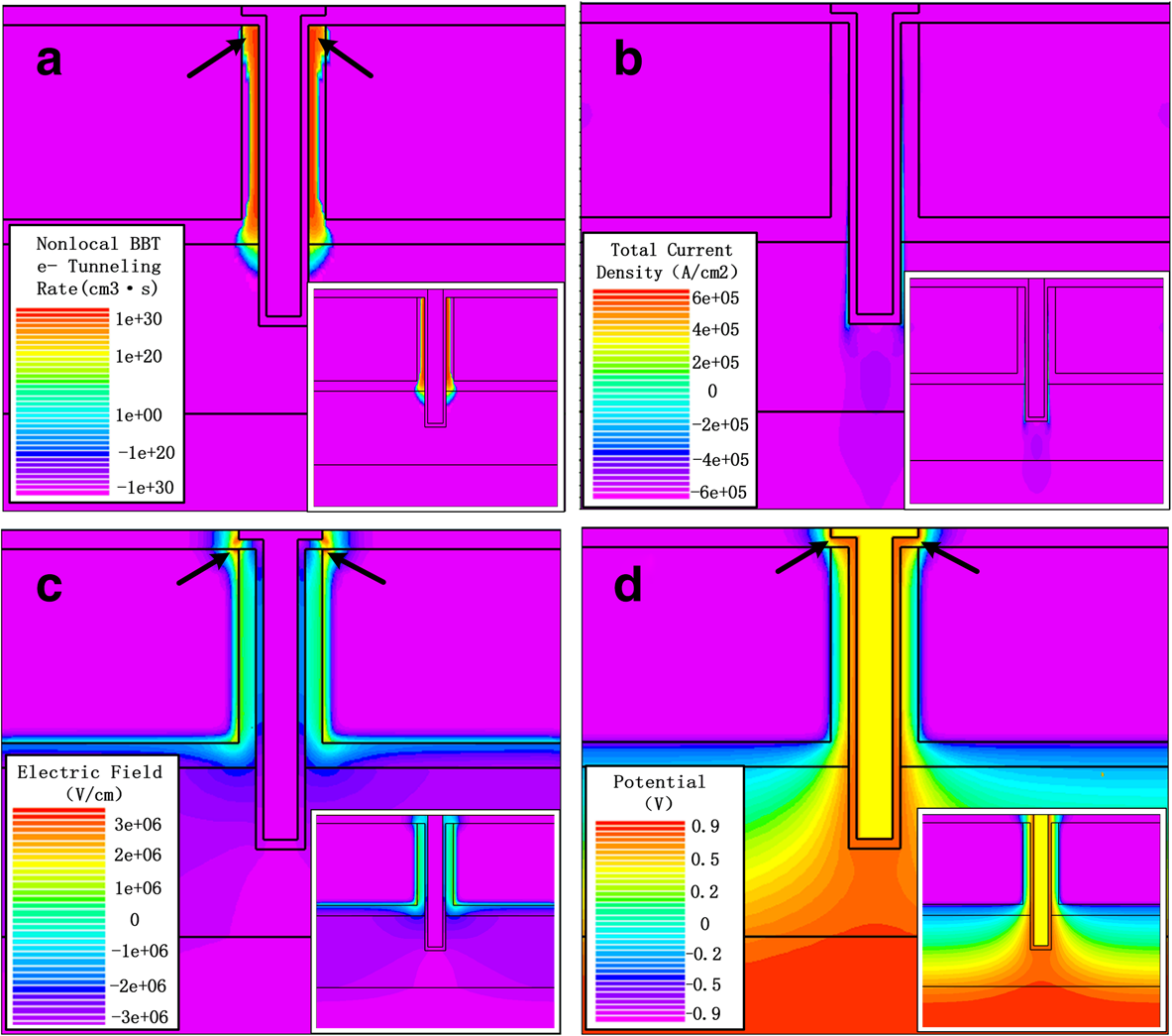
Simulated diagrams of **a** electron BTBT rate, **b** total current density, **c** electric field, and **d** potential at *V*_g_ = 0.5 V and *V*_d_ = 0.5 V

Figure 5 shows the diagrams of electron BTBT rate, total current density, electric field, and potential at *V*_g_ = 0.5 V and *V*_d_ = 0.5 V. The electron BTBT rates are shown in Fig. 5a, and the currents flowing from both gate-oxide interfaces to the drain region are shown in Fig. 5b. The gate-pocket overlap regions enhance the gate fringe electric field at the top of tunneling junctions, so the significant bending of potential contour is produced at the top of tunneling junctions, which can be seen in Fig. 5c, d. However, without the lateral gate-pocket overlap regions, the electric field at the top of tunneling junctions does not increase significantly. Therefore, the potential contour does not bend obviously, which is observed from insets in Fig. 5c, d.

**Fig. 6:**
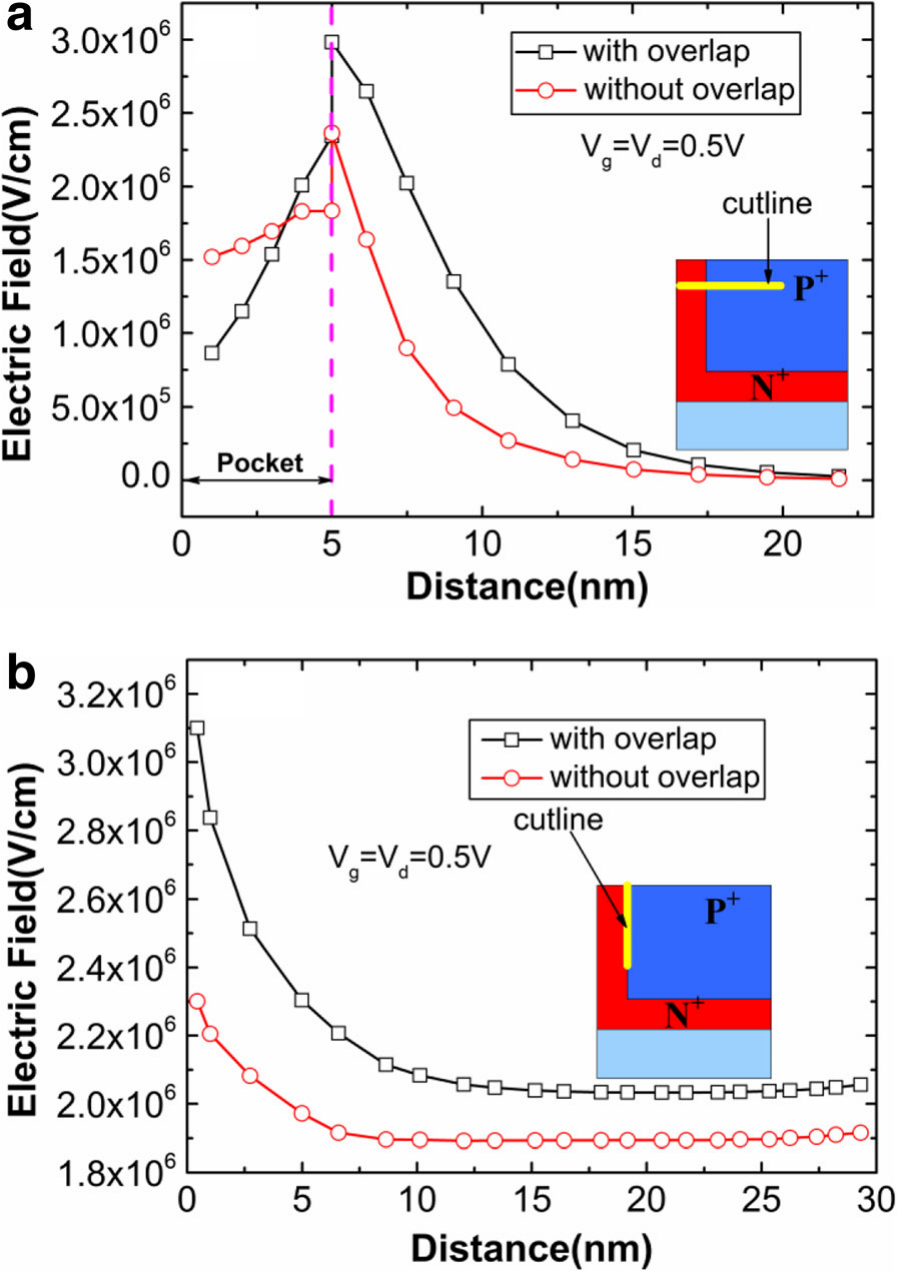
**a** Lateral and **b** vertical electric field of HTG-TFET with and without overlap

**Fig. 7:**
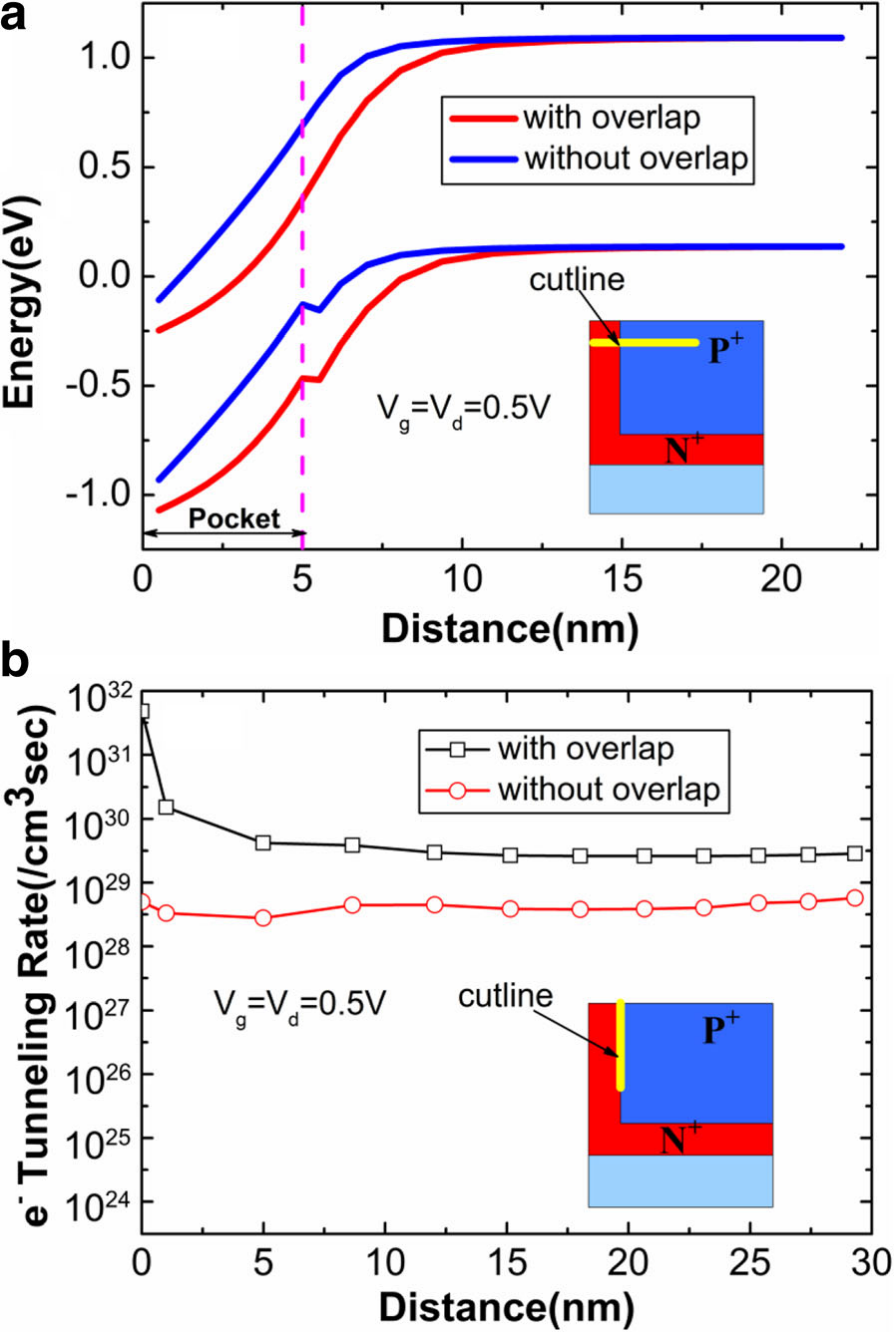
**a** Energy band diagrams of HTG-TFET with and without overlap. **b** Electron tunneling rates of HTG-TFET with and without overlap

In order to clearly understand that gate-pocket overlap regions can improve the device performance, the electric fields of HTG-TFET with and without overlap in both the lateral and the vertical directions are shown in Fig. 6. Figure 6a reveals that gate-pocket overlap enhances the electric field at the top of tunneling junction. Figure 6b reveals that the HTG-TFET with overlap also gains the greater electric field at pocket-source interface. Considering the strong dependence of energy band on the electric field, the energy band diagrams of HTG-TFET with and without overlap are plotted in Fig. 7a. Due to the enhanced electric field by the overlap, the energy band with overlap bends more sharply than that without overlap. As a result, the overlap causes the larger aligned region of energy bands, which is shown in Fig. 7a. Thus, it is likely that more electrons will tunnel in the device with overlap. This can be demonstrated by the increased tunneling area (at the top of tunneling junction) of the HTG-TFET with overlap in the Fig. 5a. Furthermore, Fig. 7b shows that overlap enhances the electron BTBT rates in the pocket regions. Both the increased tunneling area and tunneling rate are attributed to the increased of the electric field at the top of tunneling junction.

**Fig. 8:**
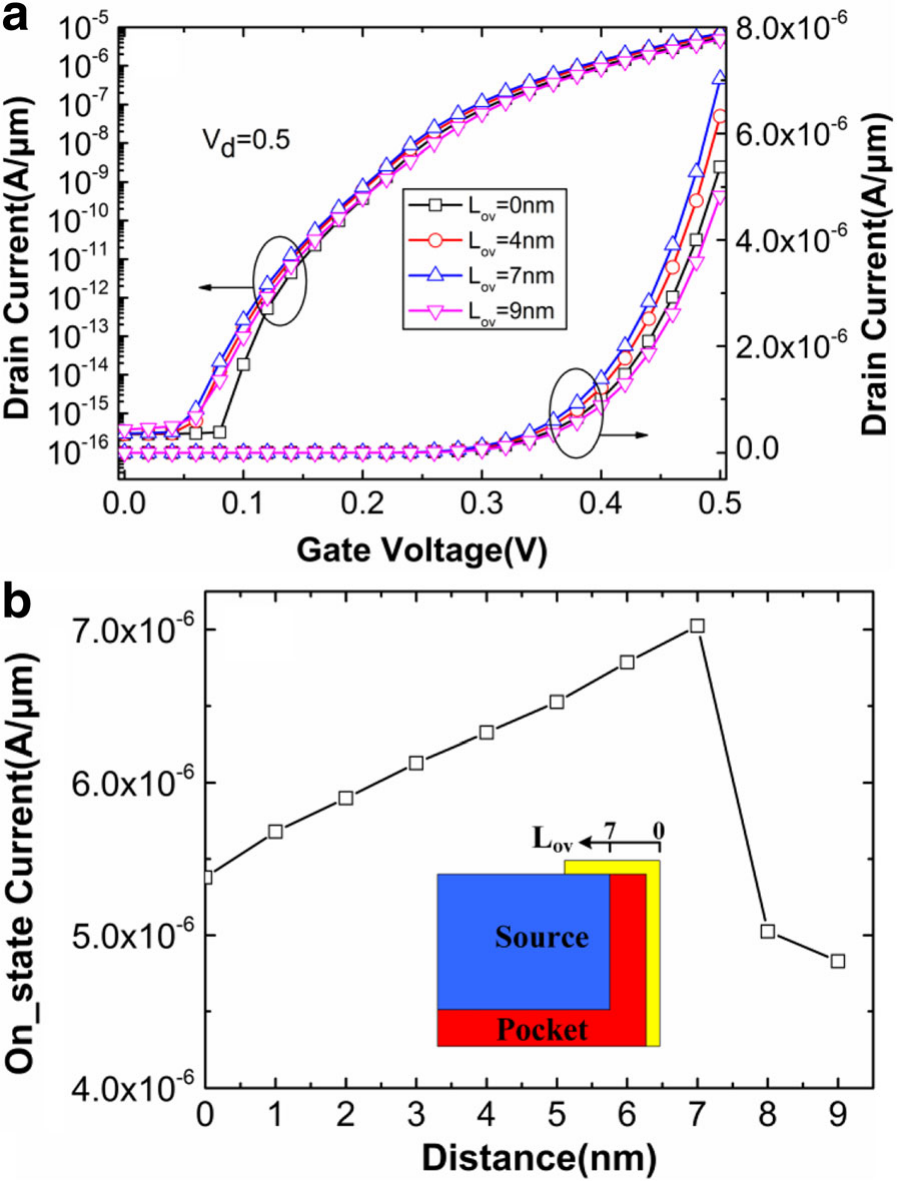
Variations of **a** transfer characteristics and **b** on-state drain current with *L*_ov_

Figure 8a, b respectively shows the variations of transfer characteristics and on-state drain current with the length of gate-pocket overlap (*L*_ov_). The comparison is conducted by keeping the same *L*_g_ and *H*_g_. The drain current increases when the *L*_ov_ increases from 0 to 7 nm. However, drain current starts to decline when the *L*_ov_ is larger than 7 nm, which means that drain current reaches the maximum 7.02 μA/μm when the boundaries of the lateral gate align with the source/pocket interfaces. When the *L*_ov_ is smaller than 7 nm, the reduction of on-state current can easily be explained by the electric field’s lowing near the tunneling junction. However, on-state current also decreases when *L*_ov_ is greater than 7 nm. This is because the electric field near the tunneling junction does not increase with the further increasing of *L*_ov_, whereas, the energy band’s lowing in the source region results in a less steeper bending [10].

**Fig. 9:**
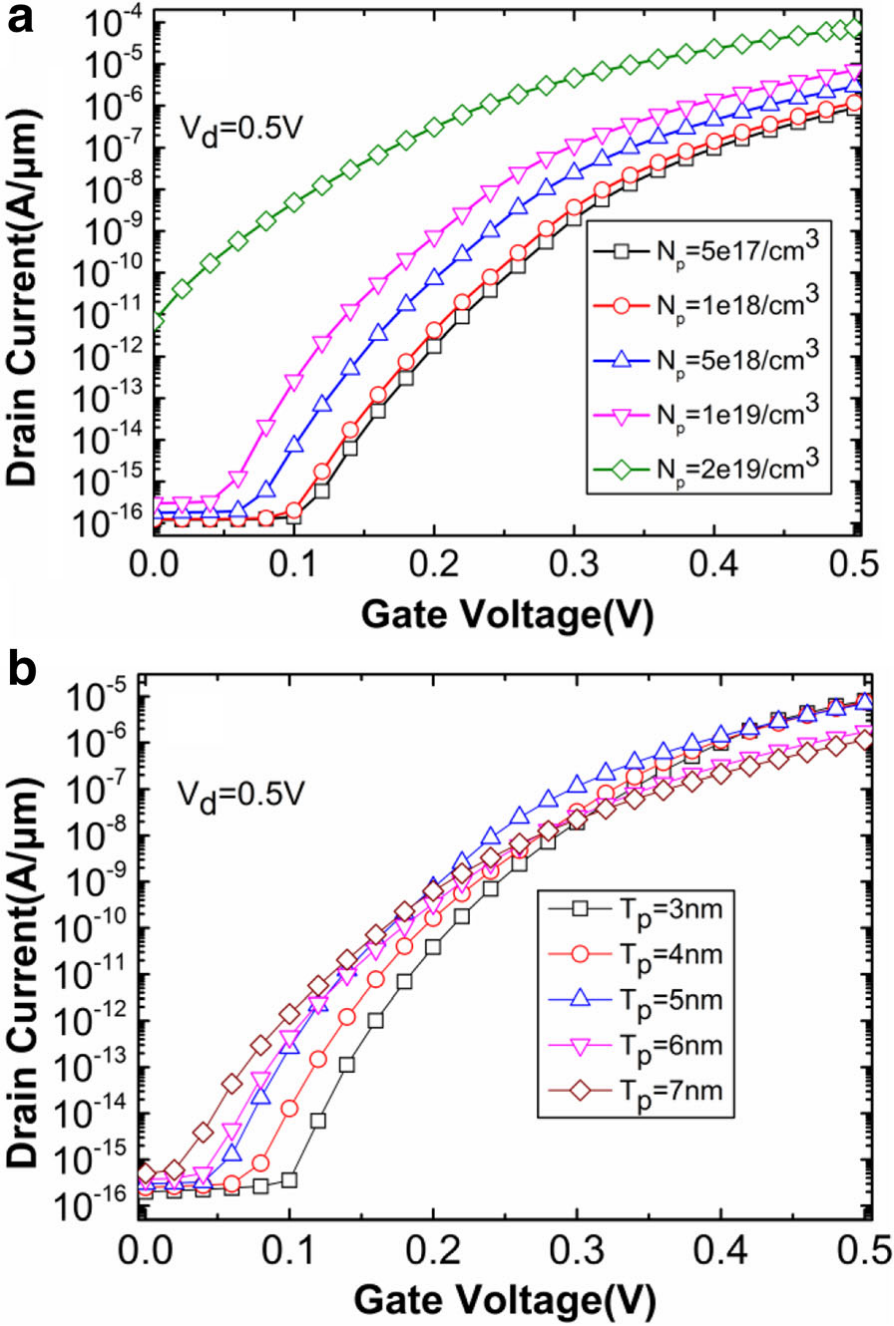
Variations of transfer characteristics with different **a** *N*_p_ and **b** *T*_p_

**Fig. 10:**
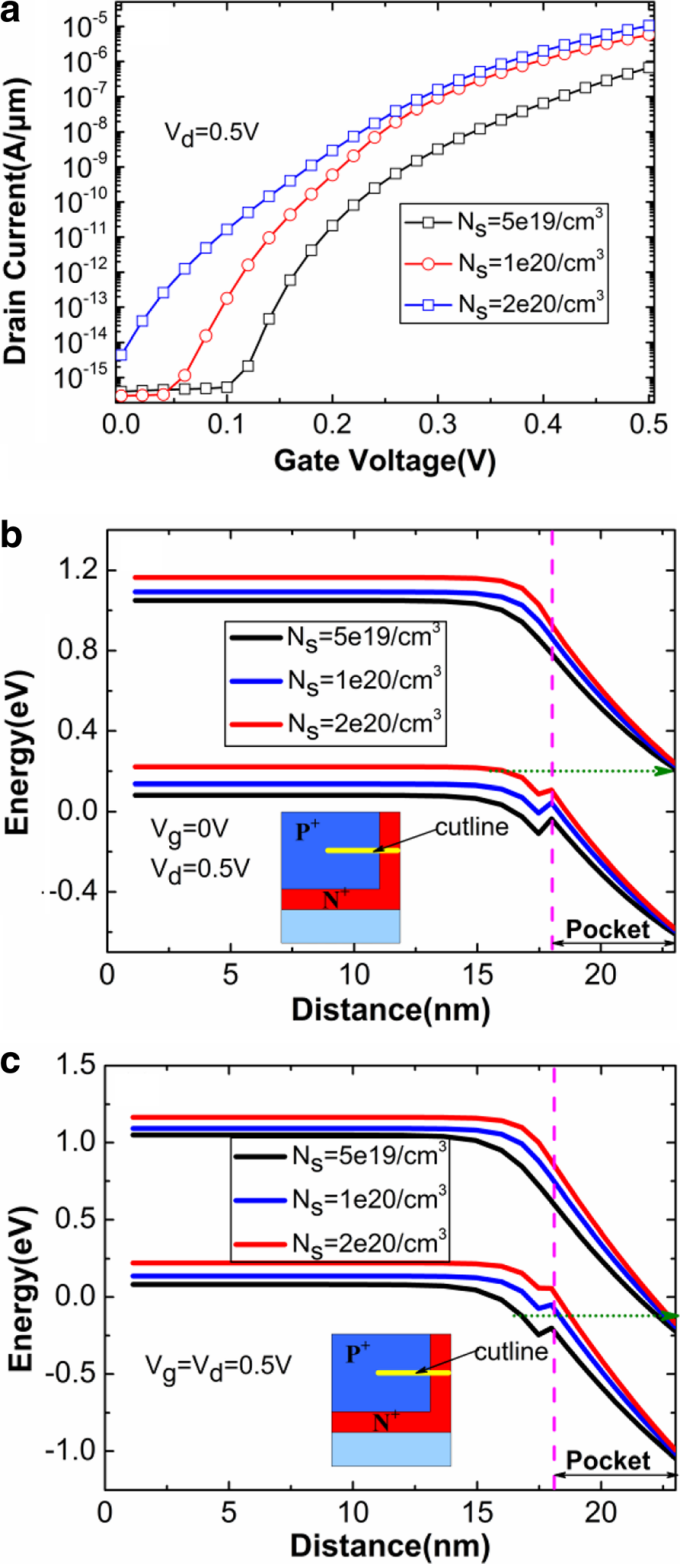
Influences of *N*_s_ on **a** transfer characteristics, **b** off-state energy band, and **c** on-state energy band

Subsequently, the main device parameters (*N*_p_, *T*_p_, *N*_s_) influencing tunneling rate are studied. The variations of transfer characteristics with different pocket-doping concentrations (*N*_p_) are shown in Fig. 9a. The off-state drain current increases by four orders of magnitude when the *N*_p_ increases from 1 × 10^19^/cm^3^ to 2 × 10^19^/cm^3^. This is because conduction band and valence band of tunneling junction closely align with each other in off-state when the *N*_p_ is very high. As a result, 1 × 10^19^/cm^3^ is regard as the optimal *N*_p_ because it obtains not only higher on-state current but also lower off-state current. The pocket thickness (*T*_p_) also has influence on the performance of HTG-TFET on condition that other parameters remain constant. Figure 9b shows that off-state current increases with increasing *T*_p_. When *T*_p_ is large, the wider depletion region leads the pocket to be partially depleted, resulting in an increased electron concentration between the source and the pocket regions. The subthreshold conduction is now determined by the carrier diffusion instead of the BTBT. Besides, the source-doping concentration (*N*_s_) has the similar influence on the device performance. From Fig. 10, it can be obviously observed that the lower *N*_s_ and higher *N*_s_ have the bad effects on on-state current and off-state current, respectively. When the *N*_s_ is 2 × 10^20^/cm^3^, the valence band pulled up significantly in the source region leads to the smaller tunneling distance at off-state, which can be seen in Fig. 10b. When the *N*_s_ is 5 × 10^19^/cm^3^, there is a very large tunneling distance at on-state, which diminishes electron BTBT rates, as shown in Fig. 10c. In conclusion, the optimal *N*_p_, *T*_p_, and *N*_s_ are chosen as 1 × 10^19^/cm^3^, 5 nm, and 1 × 10^20^/cm^3^, respectively.

Finally, the capacitance characteristics of HTG-TFET, LG-TFET, and UTFET are also investigated by using an AC small signal simulation with the operating frequency of 1 MHz. In the TFETs, due to the presence of source-side tunneling barrier, the gate-to-source capacitance (*C*_*gs*_) is very small. Therefore, the Miller capacitance mainly depends on the gate-to-drain capacitance (*C*_*gd*_) [19, 20].

**Fig. 11:**
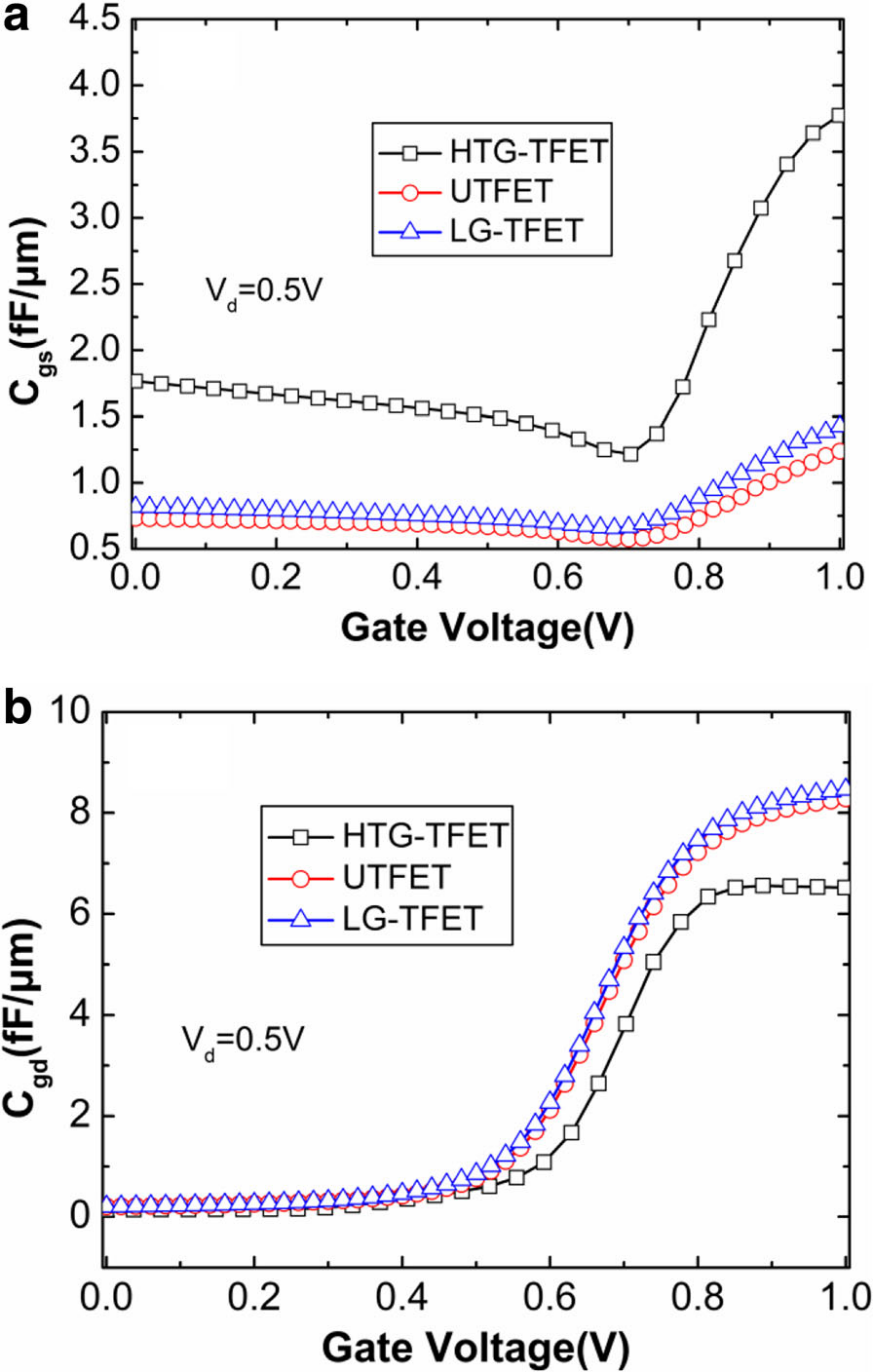
Capacitance-voltage characteristics of the HTG-TFET, UTFET, and LG-TFET at *V*_d_ = 0.5

Figure 11 shows the capacitance-voltage characteristics of the HTG-TFET, UTFET, and LG-TFET. From Fig. 11a, it can be seen that *C*_*gs*_ of HTG-TFET is larger than that of UTFET and LG-TFET. This is due to the larger overlap of *C*_*gs*_ in HTG-TFET. The inversion layer is formed from the drain region and is expanded into the source region by increasing gate voltage, which screens the source-side inner-fringe capacitance to reduce *C*_*gs*_. However, when the gate voltage is larger than 0.7 V, the elimination of inner-fringe capacitance makes the barrier capacitance across tunneling junction be dominated component of *C*_*gs*_. Therefore, *C*_*gs*_ of these three devices increases with increasing gate voltage. In these three devices, the increasing of *C*_*gd*_ with the increasing gate voltage, as shown in Fig. 11b, is mainly caused by depletion capacitance at drain side. In the HTG-TFET, although the drain is placed at the bottom of gate, the light doping channel separates the gate oxide and drain region. As a result, the *C*_*gd*_ corresponds to series capacitances of overlap capacitance and depletion capacitance, which is helpful for the suppression of *C*_*gd*_. Due to the decreased *C*_*gd*_, this design cannot cause severe Miller capacitance problem for circuit design.

## Conclusions

In this paper, a novel heterojunction TFET with a T-shaped gate (HTG-TFET) is proposed and its advantages over other counterparts are studied using Silvaco-Atlas simulation. Due to the overlap of gate and pocket in both the vertical and the lateral directions, the tunneling area and electric field at the top of tunneling junction are enhanced so that on-state drain current increase obviously. Moreover, the heterojunctions formed between silicon source and SiGe pocket regions help device to obtain better performance. Although dual sources in HTG-TFET increase *C*_*gs*_, the reduced *C*_*gd*_ cannot bring sever Miller capacitance. The parameters that affect the performance of HTG-TFET, including *L*_ov_, *N*_p_, *T*_p_, and *N*_s_, are also investigated by simulations. On the premise that optimal parameters are used in simulations, the HTG-TFET obtains the optimum performance that on-state current is 7.02A/μm, average SS is 44.64 mV/dec, and point SS is 36.59 mV/dec at *V*_g_ = 0.2 V. Therefore, HTG-TFET can be a potential candidate for the next generation of low-power electron device.
